# No mention of animal welfare in the United Nations’ sustainable development goals? But this may be a good thing!

**DOI:** 10.1017/awf.2026.10088

**Published:** 2026-05-15

**Authors:** Jacob Bull, Birte L Nielsen, Anna Silvera, Håkan Tunon, Linda J Keeling

**Affiliations:** 1 Centre for Gender Research, Uppsala University, Sweden; 2 Universities Federation for Animal Welfare, Wheathampstead, UK; 3 The Swedish Poultry Meat Association, Sweden; 4 Swedish Biodiversity Centre, Swedish Species Information Centre, Swedish University of Agricultural Sciences, Uppsala, Sweden; 5 Dept of Applied Animal Science and Welfare, Swedish University of Agricultural Sciences, Uppsala, Sweden

**Keywords:** Animal Welfare, Development, Food Production, Sustainability, SDGs

## Abstract

In this Opinion Paper, we argue that the absence of animal welfare in the United Nations’ sustainable development goals (SDGs) may not be as detrimental as some suggest. We put forward the view that the welfare of animals is an integral part of the concept of sustainability, that development which affects animals cannot be sustainable without due consideration to their welfare, and we give examples in support of this position. Put simply: no mention means animal welfare could be, and potentially should be, anywhere and everywhere in the goals. For livestock species, we submit that the synergies between the SDGs when animal welfare is included greatly outweigh the conflicts usually highlighted. Further, considering animal welfare as both an achievable goal and as a mechanism for sustainable development allows improvement of animal welfare to carry the weight it warrants: an animal with poor welfare is not a sustainable animal. By extension, products from animals with poor welfare cannot be considered sustainable, and animal welfare is necessarily included in a well-functioning ecosystem. Through the paper we argue that the challenge is not to add in animal welfare, but to think sustainability *with* animal welfare. We conclude by giving directions to where animal welfare can be integrated when developing sustainable actions.

## Introduction

The Brundtland Report (1987) definition of “*development that meets the needs of the present without compromising the ability of future generations to meet their own needs*” remains the commonly used framing of sustainable development. This definition, combined with the United Nation’s sustainable development goals (SDGs) have led to sustainability being associated with three main pillars (Purvis *et al.* 2019) relating to the environment (focused on climate, resources, and nature), society (focused on social aspects, human health, and safety), and economy (focused on efficient and sustainable use of resources). Ask the person sitting next to you on the bus what they think of when hearing the word ‘*sustainability*’, and the answers would most likely include subjects such as combatting climate change, using less plastic, and feeding a growing population rather than being about animal welfare. However, since the publication of the United Nations’ sustainable development goals in 2015, it has been a subject for discussion – and a surprise to many – that animal welfare is not mentioned (UN 2015; Keeling *et al.* 2019; Sebo *et al.* 2022; Hendriks *et al.* 2025). The goals are anthropocentric, and do not address that the well-being of humans (which is mentioned three times) is closely linked with the welfare of animals, though this link is developed in both the One Health and One Welfare concepts (WHO 2008; One Welfare 2014).

In this Opinion paper we first propose that the absence of animal welfare in the SDGs may not be such a bad thing after all, provided that we recognise the interconnection of human and animal welfare. We put forward the argument that because animal welfare is an integral and implicit part of sustainability, the lack of explicit mention is less significant. Next, we touch upon the perceived tension points between animal welfare and sustainable development, which may be fewer than expected. We then identify synergies and show that animal welfare does not always clash with other aspects of sustainability. We conclude by suggesting areas of future work and where action needs to be prioritised.

## Animal welfare – an integral part of sustainability

We are not the first to suggest that animal welfare is an integral part of sustainability (e.g. Verniers 2021; Herdioza *et al*. 2024; WOAH 2024; Keeling 2025; Hendriks *et al.* 2025), but it is easy to overlook when the other components of sustainability are more frequently centralised in the debate. Furthermore, some attempts to make the SDGs less anthropocentric fall short by focusing mainly on nature and biodiversity (e.g. Keitsch 2018) rather than the close interactions between animals and humans. Significant contributions have been made through the development of both the One Health and the One Welfare concepts. While we acknowledge the importance of critically engaging with these issues, authors such as Lindenmayer and Kaufman (2021) and Platto *et al*. (2025) argue that One Health may remain overly anthropocentric, and One Welfare may be too two-dimensional to negotiate the complex socio-ecological interactions at hand. A closer conceptual connection between animal welfare and sustainability is therefore the simplest and most logical way forward.

The development of One Health and One Welfare has occurred in a context where sustainability has been critiqued as so loosely defined that it is no longer operational. Adams (2006) argues that sustainability is “*holistic, attractive, elastic but imprecise* [and that] *the idea of sustainable development may bring people together, but it does not necessarily help them to agree goals. In implying everything, sustainable development arguably ends up meaning nothing*”. Does the addition of welfare to sustainability thus constitute a further watering down of an already overloaded concept? In the following, we argue no. Instead of proposing one unifying concept that resolves perceived tensions between traditions, we argue that animal welfare is already integral to sustainability and that the question is how to *do* sustainability *with* animal welfare.

The integration of animal welfare into sustainability is inescapable, because (non-human) animals and their welfare permeate our (human) lives – and the anthropocentric SDGs are mainly about making human lives better in a sustainable way. This cannot be achieved if the means involve avoidable suffering and distress to the animals with whom we share this planet. Moreover, this is already woven into the many ways we act, regulate, and live with animals. There are multiple examples of how the mental and physical state of animals, who are affected by human activity, is taken into consideration as part of a social licence to operate: it is inherent in the regulations surrounding the use of animals for scientific studies (EU Directive 2010), where the replace, reduce, and refine principles require the severity (i.e. likely harm) of a given treatment to be minimised as well as justified relative to the potential benefit. It is reflected in the way we think about and treat our companion animals, whom we keep despite some of them having a large carbon footprint (Su *et al.* 2018; Martens *et al.* 2019). Increasingly, human foods containing animal protein are labelled to allow the consumer to choose products that come from animals with a high level of welfare (e.g. Beter Leven; https://beterleven.dierenbescherming.nl/english/ in the Netherlands and Etiquette Bien Être Animal; https://www.etiquettebienetreanimal.fr/en/ in France). Indeed, when the welfare of wild animals is impacted by humans, efforts are made to mitigate any adverse welfare issues, both in natural ecosystems (De Ruyver *et al.* 2023; James *et al.* 2025), and in zoo design (Martínez-Macipe *et al.* 2015; Whitham & Miller 2016). Not only does human action, and therefore our attempts to develop sustainable futures, affect animals but the welfare of animals affects human well-being, health, and economies.

These are but a few examples of how animal welfare is already integrated into the way humans live, think, and act. Non-human animals therefore are in the SDGs by implication, because people are in the SDGs. The social licence to operate mentioned above is needed whenever an animal impacts or are impacted by our actions. The absence of any mention of animal welfare in the SDGs may have been an oversight, but it reflects the intertwining of animals in our social worlds and is the grounds for why their welfare is – and has to be – an integral part of sustainability.

## Potential points of conflict and synergies in achieving sustainability

Following Herdoiza *et al*. (2024), we acknowledge the absence of animal welfare from the sustainability goals can be seen as problematic and that “*This omission has led to growing criticism, as human centeredness (anthropocentrism) has failed to lead humanity towards sustainability*” (p 815). However, an explicit inclusion of animal welfare within sustainability frameworks, as suggested by some, could potentially lead to further ‘goal conflicts’ in an already tension-filled arena (Nilsson *et al.* 2016). For example, it is argued that keeping slower-growing animals in a larger space will increase the carbon footprint per kg meat yielded (Olsen *et al.* 2023) and producing fewer animals per unit area and per unit time will make the product more expensive and thus less affordable for many. Connecting to the hypothetical comment from the bus passenger earlier, there is also the issue that feeding an increasing population means that we need a secure and plentiful supply of food. However, we suggest that the language of conflict risks polarising the discussion in which sustainability and animal welfare are positioned as competing in a zero-sum game (Parlasca & Qaim 2022).

Such a zero-sum approach and focus on conflict fails to grasp the complexity of the interactions and risk stymieing progress in both arenas. Indeed, forming a separate SDG goal (Visseren-Hamakers 2020) or fourth pillar of welfare (Boyle & Stevenson 2025) risks siloing welfare and therefore increasing conflict-based logics. We recognise that there may be tensions and frictions between animal welfare and some SDGs, but there are also positive interactions and multiplier effects (Keeling *et al*. 2022; Keeling 2025). Rejecting the language of conflict does not imply that animal welfare takes priority over the other components of sustainability, but that sustainability is achieved through, by, and with, animal welfare in a complex negotiation of what it means to fare well for and with animals. This is in line with approaches where sustainability addresses both goal conflicts *and* synergies (Nilsson *et al*. 2018; for a good review of the inter-linkages among SDGs in general, see the analysis by Breuer *et al.* 2019, although – again – there is no mention of animals at all) and is not an issue specific to animal welfare.

Here, we suggest that it is helpful to differentiate between animal welfare as a goal and as a mechanism; while the aim of animal welfare science and practice is to improve the well-being of animals in a particular sector, it is also a process, constantly evolving as science, practice, and social values change. Thus, animal welfare goals operate within (multiple) SDGs aiding to their delivery. No one is arguing that animal welfare improvements in, for example the livestock sector alone, will end world hunger and provide food security, but in at least the short to medium term, it is difficult if not impossible to achieve this goal without considering animal welfare.

**Figure 1.**
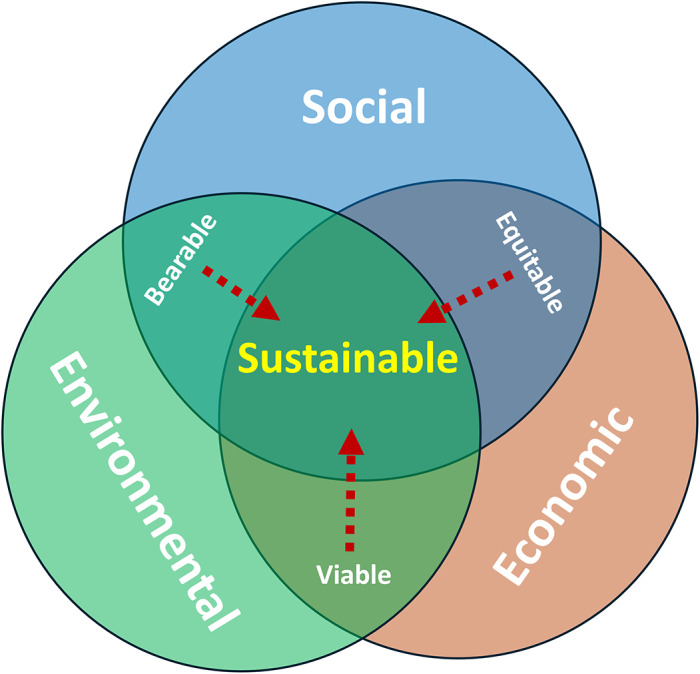
Animal welfare as a mechanism for sustainable development. The three pillars of sustainability pictured as circles overlapping in areas with sustainable solutions at the centre (adapted from Adams 2006). Whenever animal welfare is relevant, it is in the overlap between two or more circles. Improving animal welfare, as indicated by the red arrows, contributes to the move towards sustainable solutions.

Consequently, and in contrast to tensions that reflect the division of sustainability as economic, environmental, and social, we suggest that approaching animal welfare as integral to sustainability reduces conflicts (in contrast to the siloed conceptualisations that a fourth pillar would bring). In this way, animal welfare can be integrated across the three pillars of sustainability. We posit that animal welfare is never *just* economical, or *just* social, or *just* environmental. Therefore, it must be in the *overlap* between two or all of the three pillars, and contribute to moving towards a sustainable future, as indicated by the arrows in Figure 1. While in practice the interaction between animal welfare and sustainability can cut across multiple SDGs, the diagram demonstrates where animal welfare is located within the conceptual framework of sustainability as intertwined with goals and a mechanism for achieving sustainability.

The challenge thus is to think sustainability *with* as opposed to *against* animal welfare. We submit that, by highlighting areas where animal welfare and other sustainability vectors improve together, it is possible to promote *development towards* sustainability (mechanism), whilst at the same time removing siloed thinking as a reason for inactivity. Indeed, this is reflected in the increasing evidence that purchase of more expensive, high-welfare meat leads to reduced meat consumption to an extent that can have mitigating effects on the climate (Heerwagen *et al.* 2014). It has long been known that handling farm animals better during transport and slaughter improves animal welfare as well as the quality of the meat (Gregory 2008), and lower stocking densities can reduce the occurrence of injury and disease (Ellis *et al.* 2002; El Sabry *et al.* 2022; Said *et al.* 2024), potentially mitigating the risk of antimicrobial resistance. Controlling the trade of exotic pets and wildlife not only protects these animals but lowers the movement of zoonotic pathogens (Tran & Xie 2024; Aguiar *et al.* 2025) and contributes to ecological sustainability. These are but a few examples of where sustainable synergies can be found. Some of them address the connection between animal welfare and climate change, some address the link between animal welfare and human health, and they all constitute an improvement for the well-being of the animals involved. As well as highlighting the complex relationship between animal welfare, human well-being, and care for the planet, they demonstrate how animal welfare can be both a mechanism and a goal for sustainability.

## How to progress in sustainable treatment of animals

An attempt was made to rectify the absence of animal welfare in the SDGs in a resolution adopted by the United Nations Environment Assembly on 2 March 2022 (United Nations Environment Assembly 2022), in which it was acknowledged that “*animal welfare can contribute to…achieving the Sustainable Development Goals*”. This bodes well for future inclusion of animal welfare as an integral part of sustainability in revised SDGs. But change does not happen unless there are more calls for action (Sebo *et al.* 2022). That animal welfare is not named in the SDGs means that it is not siloed in a particular goal or pillar. The challenge therefore is to avoid the zero-sum calculus of conflicts and think animal welfare *with*, rather than against, sustainability. There needs to be more focus on synergies in development and concrete suggestions of how to proceed with and improve specific targets. Adding available and validated indicators of animal welfare to the existing indicators of sustainable development to monitor progress is one important step. This will promote development that is sustainable also when it comes to the animals involved, aiding this process and help change the way we think about sustainability. As mentioned, this is not without risk, but we should not let the perfect be the enemy of the good. Hopefully, the bus passenger will soon mention better treatment of animals when asked what sustainability constitutes.

For those working with animals and animal welfare this may be obvious but, for those working with other aspects of sustainability, it may not. We all need to keep reminding ourselves, and others, that animal welfare is an integral part of sustainability, and this includes all animals in human care or affected by human activity, not just farm species. To prevent polarising the discussion, we must get into the habit of defining a sustainable animal as an animal with good welfare. As we have shown, this will have benefits for humans as well as animals. In the case of animals enrolled in the production of food and other products for human consumption, the added value of animal welfare *must* be founded in real improvements to the lives of the animals concerned to prevent a welfare-washing of the concept and loss of consumer trust (Buller & Roe 2012). Together, we can ensure that animal welfare is part of sustainability frameworks. And here is (perhaps) the crux: any future development, in order to be sustainable, needs to integrate animal welfare whenever relevant, because sustainability can only be achieved if the animals we affect, keep, eat, or play with have good welfare.
